# The Influence of Parental Involvement on Parent Satisfaction: The Moderating Effect of Parental Educational Level and the Number of Children

**DOI:** 10.3389/fpsyg.2021.752802

**Published:** 2022-01-05

**Authors:** Mingming Shao, Wei He, Li Zhao, Yu-Sheng Su

**Affiliations:** ^1^School of Education Science, Nanjing Normal University, Nanjing, China; ^2^Department of Computer Science and Engineering, National Taiwan Ocean University, Keelung, Taiwan

**Keywords:** online learning, parental involvement, parent satisfaction, education level, the number of children

## Abstract

With the change in the location of school education from the classroom to the home during the COVID-19 outbreak, there should be more educational caregiving from children’s parents when children learn online. Parental involvement in children’s online learning including study guide and psychological counseling is the specific content of educational caregiving, which is different from face-to-face learning. More attention should be paid to parental involvement and parents’ satisfaction with the online learning effect. This study therefore conducted a survey on middle school students’ parents to establish a moderating model, exploring the influence of parental involvement on parent satisfaction, and the number of children and parental education level as the moderator variables influencing parental involvement. The results show that there is a significant positive correlation between parental involvement and satisfaction, while the parental education level and the number of children both have a moderating effect on the relationship between parental involvement and parent satisfaction. The moderating effect of the education level of parents shows negative, while the moderating effect of the number of children is positive. An interesting finding is that the number of children has a very low influence on parental involvement.

## Introduction

A novel coronavirus (COVID-19) is causing a worldwide outbreak (Cheng and Shan, 2020). Countries around the world have taken urgent measures in response to the pandemic, including postponing campus-based education, encouraging citizens to work and study at home, using personal protective equipment, such as masks, and canceling all large gatherings (Kickbusch and Leung, 2020). Online learning has changed the school educational location from the classroom to the home. Compared with face-to-face education, online learning requires more support, such as educational caregiving, and involvement from parents (Borup et al., 2014). Parental involvement is a key component of both traditional face-to-face and non-traditional forms of online education, including public schools, charter schools, and homeschooling (Green and Hoover-Dempsey, 2007). Russell (2004) argued that parental involvement in online education may be more important than in traditional schools. Besides, parental education level has a very important influence on parents’ involvement in their children’s learning (Krenz, 2010). The same is true for family size, specifically, the number of children (Aldosari, 2021). Parent satisfaction with online learning also reflects the quality of online education at the family level. School teachers and administrators are working hard to ensure a high-quality online education for students, but parents, as major stakeholders, may not be satisfied (Sharma and Kiran, 2021). However, there are few studies on parent satisfaction with online education.

Parents’ educational caregiving plays an important role in children’s learning, especially for young children. In the past decades, the influence of family factors on children’s learning has aroused people’s concern (Schneider et al., 2010). Family factors, such as parental education level, family economic status, and family size, influence children’s development (Cen and Aytac, 2017). Parental education level affects their children throughout their lives (Fischer, 2013) and has an indelible impact on children’s academic development (Gurung et al., 2021). Moreover, the number of families in China choosing to have more than one child has increased significantly (Black et al., 2005). Existing studies show that the number of children and family size has a strong impact on children’s educational success (Mogstad and Wiswall, 2009). While many families enjoy the happiness of having more children, they also worry about whether they have invested enough in each child when they have more than one, as lack of investment may delay their children’s education.

Besides, in the traditional learning environment, many children face a number of learning and behavioral difficulties. They do not form harmonious interpersonal relationships with their teachers and classmates, nor do they have the learning skills commensurate with their peers. This leads to a range of risks, such as academic failure, lack of close friendships, and dropping out of school (O’Shaughnessy et al., 2003). In the context of online learning, children not only have to face these learning and behavioral difficulties in the traditional learning context, but are more likely to be affected by difficulties in online learning skills, which is also a concern of this study. More support should therefore be given to children by their parents while they are learning at home.

This study aimed to explore whether parental involvement in study guide and psychological counseling affects parent satisfaction with online education at the family level. Further, it is also of great value to explore whether and how the parental educational level and the number of children as moderator variables influence parental involvement when children learn online.

## Literature Review

### Parental Involvement

There are many opportunities for parents to play a role in children’s learning. Parental involvement is an important factor in children’s learning, which is the umbrella term for many different activities, including raising children at home, assisting children with their homework, discussing with teachers, taking part in school activities, and participating in school governance (Desforges and Abouchaar, 2003). Epstein (2010) proposed a widely accepted model to explain the varying degrees of parental involvement. Four types of parental involvement were identified in her research: (1) The basic obligation of parental education; (2) communication between school and family; (3) parental involvement in the school; and (4) parents’ involvement in family learning activities (Epstein, 1987). However, in online education environments, parents pay more attention to tutoring their children throughout the process of online courses and to their children’s mental health. Liu et al. (2010) emphasized the role of parental instruction and encouragement in online learning. Parental instruction is the guidance of children’s learning, and parental encouragement is the counseling and encouragement of children’s mental level and learning motivation.

There are many studies on the parental involvement in secondary education. Fan and Chen (2001) demonstrated that parental expectations and involvement in their children’s educational achievement have a consistent and positive impact on students’ academic growth. Hill and Tyson (2009) demonstrated the importance of families, home-school relationships, and parental involvement in promoting achievement at the elementary and secondary levels.

The socioeconomic status of the family, the area where the family is located, and the parents’ level of education have different degrees of influence on parental involvement. Parents of high socioeconomic status tend to continue to pay attention to their children’s academic performance (Arens and Jude, 2017). Families with high socioeconomic status also tend to be located in developed areas and to have high levels of education. Most parents in such households have a higher level of parental involvement and see it as an investment. Additionally, students in developed areas tend to be better educated and have higher academic achievement (Thirunarayanan, 2004).

### Parent Satisfaction

The concept of satisfaction comes from customer satisfaction in an enterprise and reflects the comparison between expectations and actual results (Cardozo, 1965). Later, the study of satisfaction was extended to other areas of social life. Parent satisfaction refers to the comparison between the actual level of educational quality obtained by parents as guardians of students and the expected level of educational quality obtained by children in their school education process (Fantuzzo et al., 2006). Parent satisfaction with schools is an important factor content in school effectiveness evaluation and has become an important dimension to measure the performance of public service in basic education (Skallerud, 2011).

The content of parent satisfaction is divided into two aspects. One is parents’ evaluation of school education, including school educational resources and school management. Another is parents’ evaluation of teacher education, including teachers’ professional ethics, teaching level, and teaching process (Gibbons and Silva, 2011).

The COVID-19 outbreak has brought the research on online learning satisfaction to a climax. El-Sayad et al. (2021) explored the relationship between students’ participation and their satisfaction with online learning from the perspective of students, and showed that there is a positive impact on their online learning satisfaction. Ramón et al. (2021) have the same view. Intening (2021) explored teachers’ online learning satisfaction in terms of reaction, empathy, and reliability from the perspective of teachers. Starting from online learning itself, Eva et al. (2021) explored the influence of online learning time and task quantity on parent satisfaction. Positive relationships were found among children who were able to learn online independently. This has aroused researchers’ attention to those students who lack independent learning ability and need help from their parents. Previous studies (Jinnah and Walters, 2008; Gibbons and Silva, 2011) indicated that parental involvement has a positive predictive effect on parent satisfaction. Therefore, this study explored the relationship between parental involvement and parent satisfaction in the context of online learning. Hypothesis 1 was therefore proposed as follows:

*H1*: Parental involvement has a significant positive correlation with parent satisfaction.

### Parental Educational Level and Number of Children

There are a number of theories including the cultural capital theory that relate to the educational level of parents (Dimaggio, 1982). The early cultural socialization level of parents may leave a mark on their children’s entire life (Kraaykamp and Eijck, 2011). Several studies have focused on the effect of parents’ education on their children. For example, Ardila et al. (2005) analyzed the relationship among the types of school, public or private school, parental educational level, and children’s executive function tests performance. Fischer and Lipovská (2013) studied the impact of parental education level on children’s lifelong learning. These studies have therefore begun to pay attention to the influence of parental education level and other factors of the original family on children’s learning.

Previous studies have long pointed out the effect of family size on children’s learning. Black et al. (2005) found that a large number of children reduces the family’s available resources for each child, leading to a negative impact on children’s education. Several studies have come to the same conclusion. For example, Scott and Seifert (2010) found that children from small families had higher levels of pre-school preparation skills. Almeqdad et al. (2016) found that children from large families showed less autonomous learning behaviors than children from small families, and needed more participation and company from parents. Hence, this study introduced the relevant content of the parental education level and the number of children in the context of online learning. The above-mentioned studies proved that parental involvement is influenced by various factors, such as race, socioeconomic status, parental education level, and student gender and age (Beck et al., 2013). The participation of parents requires a large amount of time investment, so the number of children is also one of the factors that affect parental involvement (Jeynes, 2005). Accordingly, Hypotheses 2 and 3 were proposed:

*H2*: Parental education level plays a moderating role in predicting the impact of parental involvement on parent satisfaction.

*H3*: The number of children plays a moderating role in predicting the impact of parental involvement on parent satisfaction.

### Research Model

**Figure 1:**
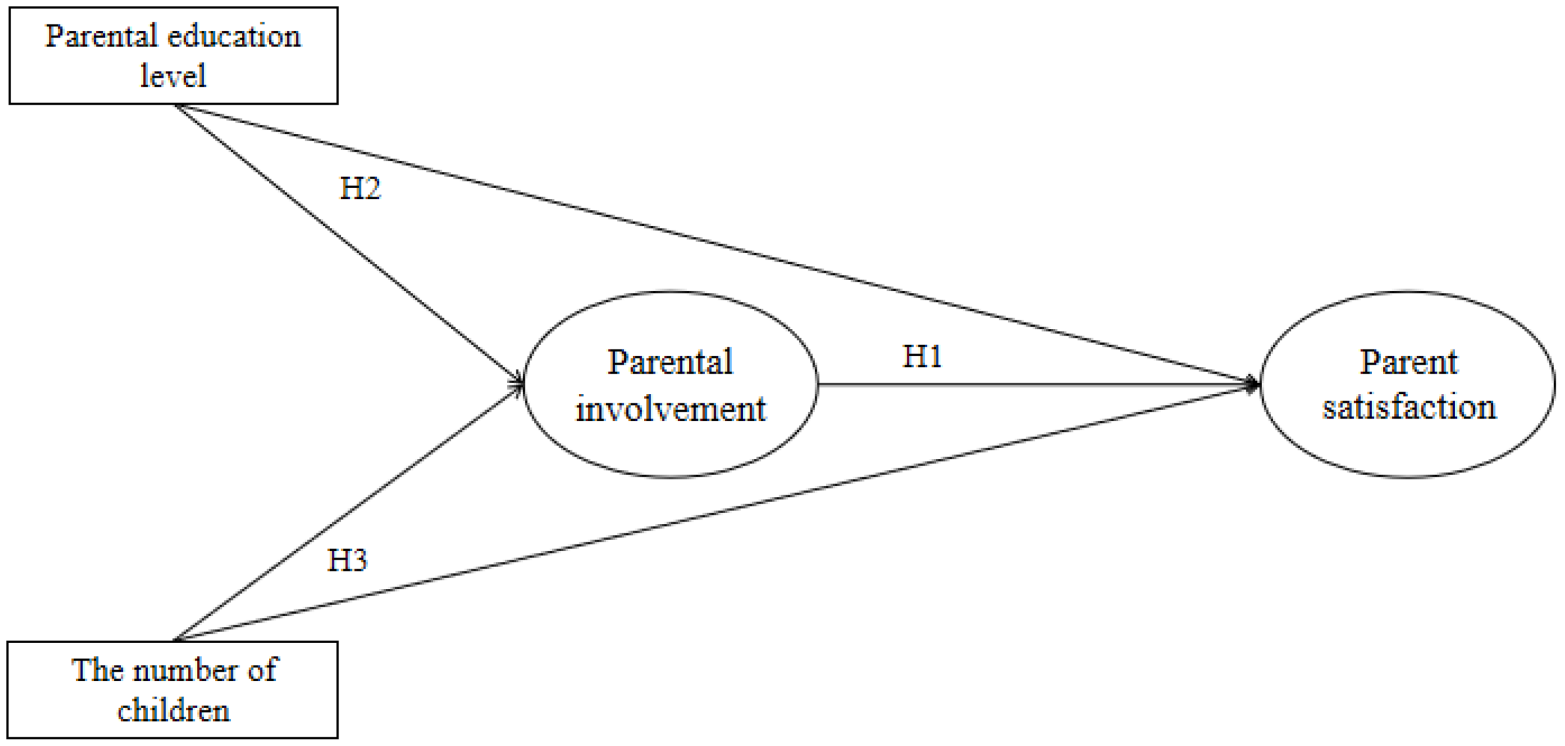
Moderating model.

The independent variable is parental involvement, and the dependent variable is parent satisfaction in the study. This study took the parental education level and the number of children as moderating variables to explore their moderating role in the prediction of parental involvement to parent satisfaction. A moderating effect model was constructed. Variables and their hypothetical relationships are shown in Figure 1.

## Materials and Methods

### Participants and Data Collection

Before the large-scale investigation, 100 test questionnaires were distributed with a recovery rate of 100%. In this study, the validity and reliability of the test questionnaire were calculated, and one item with low reliability (standardized factor load less than 0.5) was deleted.

The questionnaire was designed to be distributed online through the sample schools located in a city in the south of China. Parents were invited to finish the online questionnaire anonymously. They were informed that the results of the questionnaire would be used in a study and were not for commercial or other use. A total of 34,122 samples were collected. SPSS 24.0 was used to summarize the data, and 33,614 samples were recovered after eliminating invalid data and samples without online learning, with an effective rate of 98.5%. Among these samples, 10,909 fathers played the role of tutor in the family, accounting for 32.5% of the total, there were 22,312 mother tutors, accounting for 66.4%, 163 grandparent tutors, accounting for 0.5%, and 230 other tutors, accounting for 0.7%. In terms of regions, 25,549 (76.0%) children attended schools in cities, 6,565 (19.5%) in townships, and 1,500 (4.5%) in rural areas. The participants’ profiles are shown in Table 1.

**Table 1:** Descriptive statistics.

Item	Option content	Frequency	Percentage
Grade	1st	12,669	37.7
	2nd	11,460	34.1
	3rd	9,485	28.2
Type of school	Public schools	29,597	88.0
	Private schools	4,017	12.0
Regions of schools	City	25,549	76.0
	Town	6,565	19.5
	Rural	1,500	4.5
Role of the parent	Father	10,909	32.5
	Mother	22,312	66.4
	Grandparents	163	0.5
	Other	230	0.7
Age of the parent	Below 30	236	0.7
	31–35	2,158	6.4
	36–40	14,305	42.6
	41–45	12,785	38.0
	Above 46	4,130	12.3
Parental education level	Middle school	10,160	30.2
	High school	10,970	32.6
	College or university (4 years or less)	11,231	33.4
	College or university (more than 4 years)	1,253	3.7
The number of children	One child	20,696	61.6
	Two children	11,684	34.8
	More than two children	1,234	3.7

College or university (4 years or less) includes undergraduate and specialist education; College or university (more than 4 years) includes graduate education, which is the highest level of education.

The comparison of each dimension region is shown in Table 2. Surprisingly, the degree of parental involvement is very high, all exceeding 4. Parents are more involved in tutoring children’s learning than counseling children’s psychological status. Moreover, parents are also highly satisfied with online learning. They are more satisfied with teachers than schools.

**Table 2:** Comparison of each dimension region.

Dimension	Content	Average	SD
Parental education level		2.21	0.880
The number of children		1.42	0.563
Parental involvement	Study guide	4.40	2.997
	Psychological counseling	4.24	2.307
	Total score	4.25	5.055
Parent satisfaction	Teacher	4.15	3.221
	School	3.97	2.545
	Total score	4.07	5.537

### Instruments

By combining and summarizing the relevant literature and referring to the authoritative scale, the questionnaire was adapted and improved after discussion with relevant expert groups. It was composed of four parts: the questionnaire explanatory information; the participants’ basic information survey (four questions); the main part, including parental education level (one question), the number of children (one question), parental involvement (10 questions), and parent satisfaction with online education (10 questions). Since one item was deleted after the trial test questionnaire was collected, there were 10 questions of parental involvement in the large-scale survey. Hence the questionnaire had a total of 26 questions.

#### Parental Involvement

The independent variable in this study was parental involvement. This study drew on the parental involvement scale of Gürbüztürk and Şad (2010), and studies which adopted it to suit the online learning background of middle school students. It included 10 questions, with five on study guide and five on psychological counseling. A five-level Likert scale was used to encode parental involvement, from strongly disapprove to strongly approve as 1–5. A higher score indicates more parental involvement in their children’s online learning, while a lower score indicates less parental involvement.

#### Parent Satisfaction

The dependent variable of this study was parent satisfaction. The scale developed by Gibbons and Silva (2011) was adapted to fit the background of online learning, and included five questions on satisfaction with teachers and five on satisfaction with schools, giving a total of 10 questions. A five-level Likert scale was used to encode online education satisfaction from strongly disapprove to strongly approve as 1–5. The overall score of online education satisfaction was the sum of teacher satisfaction and school satisfaction. The higher the score, the better the online education satisfaction of parents, while the lower the score, the worse their online education satisfaction.

#### Parental Education Level

One of the moderating variables in this study was parental educational level. Parents with a middle school education were coded as 1, high school education as 2, college or university education (4 years or less) as 3, and college or university education (more than 4 years) as 4. The higher the score, the higher the parents’ education level, while the lower the score, the lower their education level.

#### The Number of Children

One of the moderating variables in this study was the number of children in the family. A family with one child was coded as 1, 2 children was coded as 2, and more than 2 children was coded as 3. The higher the score, the more children there were in the family, while the lower the score, the fewer children there were.

### Data Analysis

In this study, AMOS 25.0 was used to test the structural equation model. Process 3.0 was used to test the Bootstrap moderating effect, exploring the correlation between parental involvement and parent satisfaction. Furthermore, the moderating effects of parental educational level and number of children on parental involvement and satisfaction were examined.

## Results

### Reliability and Validity of the Instrument

AMOS 24.0 and SPSS 24.0 software were utilized to analyze the reliability and validity of the questionnaire in the study.

In order to ensure the validity of the model, the absolute and relative fitting index of the model should meet the standard (standardized factor load >0.5; RMSEA < 0.08; GFI, AGFI, NFI, and CFI > 0.9 respectively; Anderson et al., 1988; Foster et al., 2006; Hair et al., 2014). The original questionnaire had 22 items, including the education level, the number of children, parental involvement, and parent satisfaction. To analyze the scale, first-order confirmatory factor analysis was applied. Unreasonable items were deleted. If the standardized factor load of the item is less than 0.5, it should be deleted. After deleted some items, the model should meet the relevant requirements of the fitting index. In this study, the value of RMSEA was 0.074. The values of GFI, AGFI, NFI, and CFI were 0.933, 0.909, 0.956, and 0.957, respectively, indicating that the validity of the questionnaire and the fitness of the model were good. Therefore, 16 items were retained for further analysis, including one item for parental education level, one for the number of children, seven for parental involvement, and seven for parent satisfaction.

The reliability of the questionnaire needs to meet the criteria (Cronbach’s *α* > 0.7; CR > 0.7; AVE > 0.5; Cronbach, 1951; Fornell and Larcker, 1981; Hair et al., 2014). Cronbach’s *α* was used as the index of reliability analysis. The higher its value, the higher the reliability consistency of the scale. The Cronbach’s *α* of this questionnaire was 0.962 in this study, indicating excellent reliability of the questionnaire. In order to more accurately measure the reliability of the questionnaire, the composite reliability (CR) value was referred to for further measurement of the Likert scale. As shown in Table 3, the value range of CR in this study was 0.9327 and 0.949 which is greater than 0.7, indicating that the combined reliability of this model’s perspectives was excellent. This study referred to the average variance extraction values (AVE) to test the validity of the model. The AVE of all dimensions must be greater than 0.50 for the scale to be effective. The value range of AVE in this study was 0.6652 and 0.7275, indicating that this model was effective.

**Table 3:** Reliability of the model.

Latent variable	Critical value	FL	CR	AVE	*α*
Parental involvement	Study guide 1	0.720	0.9327	0.6652	0.930
	Study guide 3	0.867			
	Study guide 4	0.818			
	Study guide 5	0.868			
	Psychological counseling 1	0.781			
	Psychological counseling 4	0.856			
	Psychological counseling 5	0.788			
Parent satisfaction	To teacher 1	0.861	0.949	0.7275	0.947
	To teacher 3	0.894			
	To teacher 4	0.909			
	To teacher 5	0.909			
	To school 1	0.745			
	To school 2	0.780			
	To school 5	0.858			

CR, composite reliability; AVE, average variance extracted.

In the test of structural reliability, firstly for each dimension, the square root of AVE should be tested. As shown in Table 4, according to Schumacker and Lomax (2016), the value of square root of the AVE of all of the variables exceeded the absolute value of the correlation coefficient, indicating that this model had good discriminative validity.

**Table 4:** Validity of the model.

Construct	1. Parental involvement	2. Parent satisfaction
1. Parental involvement	**0.816**	
2. Parent satisfaction	0.567^**^	**0.853**

The bold diagonal is the square root of AVE; Non-diagonal data are Pearson correlation values.

** *p* < 0.01.

### Path Analysis

The fit index was used to evaluate the degree of fit of the model in the study. In terms of absolute fit index, the RMSEA was 0.045 (<0.08; Anderson et al., 1988) the goodness of fit index (GFI) and adjusted goodness of fit index (AGFI) were 0.926 and 0.900 (>0.9 and <1.0; Foster et al., 2006). In terms of relative fit index, the normalized fit index (NFI) and comparative fit index (CFI) were 0.951 and 0.952 (>0.9; Hair et al., 2014) while the incremental fit index (IFI) and relative fit index (RFI) were 0.952 and 0.942 (>0.9). Besides, in terms of streamlining the fit indexes, the PNFI and the PGFI were 0.801 and 0.687 (>0.5), illustrating that the model of this study fit the data well.

Path analysis was to test the research model hypotheses. All the three hypotheses were proved (see Table 5), and significant states were identified for all of the hypotheses. All the values of *p* were less than 0.001 except for NC and PS. PI had a direct positive association with PS (*β* = 0.647, *t* = 105.376***), while PEL and NC had direct negative associations with PI (*β* = −0.018, *t* = −3.995***; *β* = −0.014, *t* = −2.005*). Besides, PEL had a direct negative association with PS (*β* = −0.056, *t* = −14.211***), while NC had a direct positive association with PS (*β* = 0.006, *t* = 8.432***).

**Table 5:** Path coefficient analysis.

Hypothesis	Causal factors	Standardized coefficient (*β*)	SE	*t*	*p*
H1	PS←PI	0.647^***^	0.006	105.376	<0.001
H2	PI←PEL	−0.018^***^	0.004	−3.995	<0.001
	PS←PEL	−0.056^***^	0.004	−14.211	<0.001
H3	PI←NC	−0.014^*^	0.007	−2.005	<0.05
	PS←NC	0.052^***^	0.006	8.432	<0.001

*** *p* < 0.001;

* *p* < 0.05.

**Figure 2:**
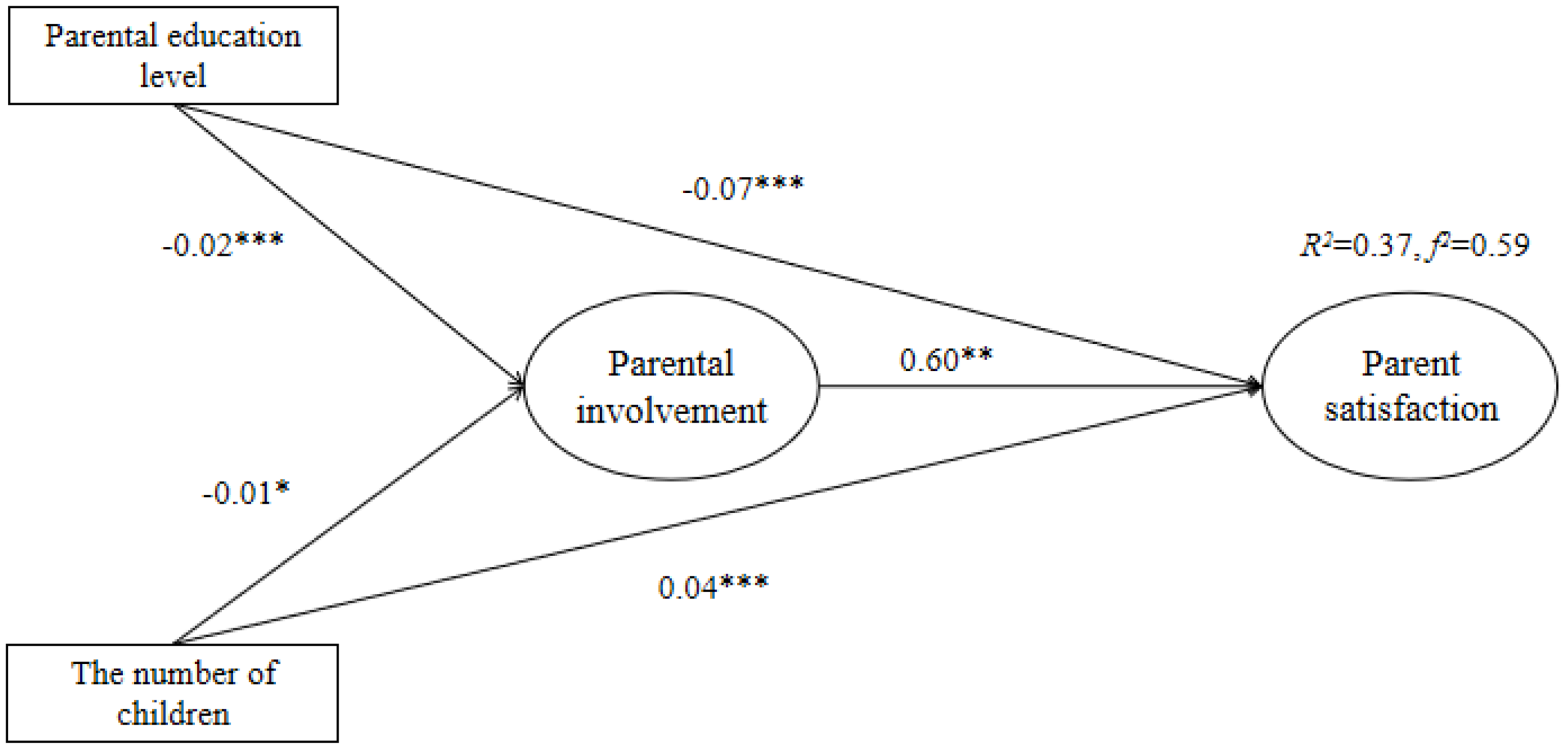
The verification of the research model. ^***^p < 0.001; ^**^p < 0.01; and ^*^p < 0.05.

The determination coefficient *R*^2^ is important for summarizing biological benefits, as the variance ratio is interpreted by the statistical model (Nakagawa et al., 2017). According to Sanchez and Golding (2013), when *R*^2^ values are less than 0.6, 0.3–0.6 is considered as medium, while less than 0.3 is considered as low. Additionally, Cohen (1988) proposed the model effect size (*f*^2^) to make sure researchers could move from recognizing statistical significance to providing a more general quantifiable description of the effect size (Kühberger et al., 2014). When the *f*^2^ value exceeds 0.8, it can be considered as large, between 0.2 and 0.8 as medium, and below 0.2 as small. According to Hair et al. (2014), the explanatory power of PI, PEL, and NC on PS in the study was 37% (*R*^2^ = 0.37, *f*^2^ = 0.59), showing good predictive power. However, the model was adjusted to improve the degree of fitting, as shown in Figure 2.

### Moderating Effect Test

This study used Model 2 in the SPSS macro compiled by Hayes (2012) to test the moderating effect of education level and the number of children on the relationship between parental involvement and parent satisfaction. The results are shown in Table 6.

**Table 6:** Standardized bootstrap moderating effect test.

Outcome variable	Predictor variable	*R*	*R* ^2^	*F*(df)	Coefficient	SE	*t*
Parent satisfaction		0.5736	0.3290	3296.2226			
	Parental involvement				0.6195	0.0049	126.4933^***^
	Parental education level				−0.4053	0.0291	−13.9487^***^
	Parental involvement × parental education level				−0.0217	0.0057	−3.7995^***^
	The number of children				0.3709	0.0454	8.1771^***^
	Parental involvement × the number of children				0.0189	0.0089	2.1160^*^

*** *p* < 0.001;

* *p* < 0.05.

Both the product of parental involvement and education level and the product of parental involvement and the number of children had significant predictive effects on parent satisfaction (parental involvement × parental education level: *β* = −0.0217, *t* = −3.7995, *p* = 0.0001; parental involvement × the number of children: *β* = 0.0189, *t* = 2.1160, *p* = 0.0343), indicating that education level and the number of children can play a moderating role in the direct prediction of parental involvement to parent satisfaction.

In this study, the moderating effect was further analyzed by simple slope (McElhaney and Allen, 2010). The result shows the different roles of parents with low education, parents with high education, families with more children, and families with fewer children in the moderating effect. For those subjects with low education level (M-1SD), parental involvement had a significant positive predictive influence on parent satisfaction (*β* = 0.6385, *t* = 91.5436, *p* < 0.001). For those subjects with higher education level (M + 1SD), parental involvement also had a positive predictive effect on parent satisfaction, but its predictive effect was smaller (*β* = 0.6004, *t* = 85.1888, *p* < 0.001) than that for parents with low education level, indicating that with the increase of parental education level, the predictive effect of parental involvement on parent satisfaction gradually decreases. That is to say, the moderating effect of educational background is higher among parents with low educational backgrounds. For subjects with a small number of children (M-1SD), parental involvement had a significant positive predictive effect on parent satisfaction, and its predictive effect was small (*β* = 0.6115, *t* = 97.8712, *p* < 0.001). However, for subjects with more children (M + 1SD), parental involvement had a positive predictive effect on parent satisfaction (*β* = 0.6301, *t* = 90.0500, *p* < 0.001), indicating that with the increase in the number of children in a family, the predictive effect of parental involvement on parent satisfaction gradually increased. In other words, in families with more children, the moderating effect of the number of children was higher.

## Discussion

The study was conducted during the COVID-19 outbreak. After a brief period of stability, the epidemic is once again sweeping the world. Online learning, as the main mode of education in emergency education, is still valued. According to the results of the above analysis, the more involved parents are in their children’s online learning, the more satisfied they will be with online education. Previous studies had the same view (Khajehpour and Ghazvini, 2011; Antony-Newman, 2019). On the one hand, the lower the degree of parental involvement, the less understanding of online learning. Due to the sudden change in educational location, parents who are not familiar with online education think that schools and teachers may not pay enough attention to their children compared with face-to-face education, which leads to parents’ low evaluation of online learning. On the other hand, the quality of online learning is largely determined by family factors. Parental tutoring and help can affect the quality of online learning, suggesting that online learning is heavily dependent on parental support and involvement. Therefore, Hypothesis 1 was proved.

Besides, the education level of parents had a negative moderating effect on parental involvement and satisfaction, indicating that parents with higher education levels are less involved in tutoring their children compared with parents with lower education levels. However, some scholars believe that there is no significant correlation between parental educational level and their participation (Vellymalay, 2010; Alghazo, 2014). The COVID-19 outbreak has forced parents to spend a great deal of time involving in children’s online learning. However, it is difficult for well-educated parents to find a balance between their heavy workload and involving in children’s online learning (Ribeiro et al., 2021), which is contrary to previous research which found that the more educated parents are, the higher their level of participation (Eccles and Harold, 1996). Parents with higher education levels have a lower evaluation of online education. Saleh and Sanders (2014) had the same view. Higher educated parents are busier with their work, which leads to less attention to their children’s schoolwork and mental health in online learning contexts. Parent satisfaction reflects the comparison between expectations of educational quality and the actual achieved results. The higher the education level, the higher parents’ expectations of online education, causing low satisfaction with online education. Hypothesis 2 was therefore supported.

A very interesting phenomenon is that there is a weak negative correlation between the number of children and parental involvement, indicating that there is no significant difference in parental involvement between one-child families and families with multiple children. This is contrary to Scott and Seifert (2010) study. Many families are worried that they will not be able to invest enough in their children’s education when they have more than one child. However, according to the data analysis, the number of children had no significant effect on parental involvement. Moreover, parental involvement in families with more children had a higher predictive effect on parent satisfaction. This is the innovation of this paper. Hypothesis 3 was thus supported.

## Conclusion

Based on 33,614 middle school students’ parents, this study explored the influence of parental involvement on parent satisfaction in the context of online learning, and whether and how parental educational level and number of children as moderator variables influence parental involvement when children learn online.

The results showed that there was a significant positive correlation between parental involvement and satisfaction (*β* = 0.647, *p* < 0.001). Parental education level and the number of children have a moderating effect on the relationship between parental involvement and parent satisfaction. The education level of parents had a negative moderating effect (*β* = −0.0217, *p* < 0.001), while the number of children had a positive moderating effect (*β* = 0.0189, *p* < 0.05). An interesting finding is that the number of children had a very low influence on parental involvement.

In addition, the moderating effect of the education level on the relationship between parental involvement and parent satisfaction was higher in the group of parents with low education levels (*β* = 0.6385, *p* < 0.001) than those with high education levels (*β* = 0.6004, *p* < 0.001). The moderating effect of the number of children in families with more children on the relationship between parental involvement and parent satisfaction (*β* = 0.6301, *p* < 0.001) was higher than in families with fewer children (*β* = 0.6115, *p* < 0.001).

### Implications

This study theoretically proves that parental involvement can significantly positively predict parent satisfaction in the online education environment, and this prediction is moderated by family background, which opens a new horizon for future research.

On a practical level, this study proves that parents with higher education have lower parental involvement and higher expectations for online learning effects, which leads to lower parent satisfaction with online learning. As online education is a new form of education, there must be some problems. The quality of online education needs the efforts of schools, families, and society. Therefore, highly educated parents should have moderate expectations and pay more attention to and help their children in online education. Parents with lower education levels should also learn more about online learning and give their children whatever support they can, especially those who are not strong in autonomous learning. One interesting finding was that the moderating effect of the number of children was very low. Therefore, parents need not worry too much about the influence of more children on other children’s education, nor should they neglect the tutoring and support of their children because of the large number of children.

In general, online education has its advantages and strengths. Parents who can provide help for children tend to be satisfied with online learning. In their views, teachers can effectively answer students’ questions on time through a variety of channels. The overall deployment of school is correct, and the learning process and progress of children can be monitored in real time. However, for parents who have no time or ability to help their children, online learning is not considered satisfactory. Therefore, it is important not only to improve the quality of online education at the levels of schools and teachers, but also the guidance of parents at the family level. For example, it is essential to choose a quiet, clean, independent space with appropriate lighting and temperature, equipped with good performance hardware devices for online learning, such as TV, computer, tablet, and mobile phone. Besides, parents should remind children to take notes and finish their coursework. They should also provide guidance for their online learning if possible. In terms of children’s mental health, parents should not give their children too much pressure. During the busy online learning, they can take their children out for a walk, play sports, etc.

### Limitations and Future Study

Some limitations should be acknowledged. The sample was from the same city. More samples from different and larger areas should be considered in the future. Additionally, previous studies on parental involvement were mostly focused on traditional learning styles. The study adapted the variable of parental involvement to make it suitable for online learning environments, which requires further research on parental involvement and parent satisfaction in the context of online learning. Finally, other variables, such as the cultural differences and socioeconomic conditions of the family, may be considered to be explored in future studies.

## Data Availability Statement

The original contributions presented in the study are included in the article/supplementary material, further inquiries can be directed to the corresponding author.

## Ethics Statement

Ethical review and approval were not required for the study on human participants in accordance with the local legislation and institutional requirements. Written informed consent for participation was not required for this study in accordance with the national legislation and the institutional requirements.

## Author Contributions

MS, WH, LZ, and Y-SS contributed equally to the conception of the idea, implemented and analyzed the experimental results, wrote the manuscript, and read and approved the final manuscript. All authors contributed to the article and approved the submitted version.

## Funding

This study was supported by the Ministry of Science and Technology, Taiwan, under grant MOST 109-2511-H-019-004-MY2 and MOST 109-2511-H-019-001.

## Conflict of Interest

The authors declare that the research was conducted in the absence of any commercial or financial relationships that could be construed as a potential conflict of interest.

## Publisher’s Note

All claims expressed in this article are solely those of the authors and do not necessarily represent those of their affiliated organizations, or those of the publisher, the editors and the reviewers. Any product that may be evaluated in this article, or claim that may be made by its manufacturer, is not guaranteed or endorsed by the publisher.
